# A 10-week remote monitoring study of sleep features and their variability in individuals with and without ADHD

**DOI:** 10.1186/s12888-025-06729-z

**Published:** 2025-03-27

**Authors:** Hayley Denyer, Ewan Carr, Qigang Deng, Philip Asherson, Andrea Bilbow, Amos Folarin, Madeleine J Groom, Chris Hollis, Heet Sankesara, Richard JB Dobson, Jonna Kuntsi

**Affiliations:** 1 Social, Genetic and Developmental Psychiatry Centre, Institute of Psychiatry, Psychology and Neuroscience, King’s College London, 16 De Crespigny Park, London, SE5 8AF UK; 2 The Department of Biostatistics and Health Informatics, Institute of Psychiatry, Psychology and Neuroscience, King’s College London, London, UK; 3 The National Attention Deficit Disorder Information and Support Service, ADDISS, Edgware, Middlesex UK; 4 Institute of Health Informatics, University College London, London, UK; 5 NIHR Biomedical Research Centre at South London and Maudsley NHS Foundation Trust and King’s College London, London, UK; 6 Health Data Research UK London, University College London, London, UK; 7 NIHR Biomedical Research Centre at University College London Hospitals NHS Foundation Trust, London, UK; 8 School of Medicine, Mental Health & Clinical Neurosciences, Institute of Mental Health, University of Nottingham, Nottingham, UK; 9 Institute of Mental Health, NIHR MindTech Healthcare Technology Co-operative, University of Nottingham, Nottingham, UK

**Keywords:** ADHD, Attention deficit hyperactivity disorder, Sleep variability, mHealth, Remote measurement technology, Wearable devices

## Abstract

**Background:**

People with attention deficit hyperactivity disorder (ADHD) often report disturbed sleep, as well as co-occurring symptoms of anxiety and depression. Yet studies employing objective assessments often do not show as many sleep disturbances compared to subjective measures. These discrepancies may relate to subjective reports capturing problematic nights, which may not be captured in a single night’s sleep or by averaging objective measurements over several nights. Given that variability in behaviours is in general strongly linked to ADHD, individuals with ADHD could have greater sleep variability than individuals without ADHD. Using active and passive remote monitoring, we investigate differences in the level and variability of daily sleep behaviours between individuals with and without ADHD and explore if sleep is associated with changes in anxiety and depressive symptoms across a 10-week remote monitoring period.

**Methods:**

Forty individuals (20 with ADHD, 20 without) took part in a 10-week remote monitoring study. Active monitoring involved participants completing questionnaires on ADHD and co-occurring psychiatric symptoms at weeks 2, 6 and 10. Passive monitoring involved participants wearing a wearable device (Fitbit) that measured sleep each night.

**Results:**

Individuals with and without ADHD were similar in the levels of sleep recorded each night. However, compared to those without ADHD, participants with ADHD had more variable sleep duration, sleep onset and offset, and sleep efficiency over 10 weeks. Within-individual associations of co-occurring anxiety and depressive symptoms with the sleep features were non-significant.

**Conclusions:**

In a 10-week remote monitoring study of sleep using a wearable device, we show that what distinguishes individuals with ADHD from those without is their greater variability in sleep features: participants with ADHD had a more variable sleep duration, sleep onset and offset, and sleep efficiency. Inconsistency and high variability are hallmarks of ADHD, and we show that this characteristic extends also to sleep among adolescents and adults with ADHD.

**Trial registration:**

Clinical trial number: not applicable.

**Supplementary Information:**

The online version contains supplementary material available at 10.1186/s12888-025-06729-z.

## Introduction

Sleep problems, such as difficulties initiating and maintaining sleep [1], affect up to 72% of adolescents and adults with attention deficit hyperactive disorder (ADHD) [2, 3]. Several explanations have been put forward as to why adolescents and adults may experience sleep difficulties. Hvolby et al. [4] proposed that (i) core symptoms of ADHD, such as restlessness in the evening and poor organisation, contribute to difficulty with sleep, (ii) inadequate sleep may lead to ADHD-like symptoms, or (iii) both ADHD and sleep difficulties co-occur, exacerbating each other.

Studies focussing on sleep problems in individuals with ADHD have used either subjective reports, such as questionnaires or sleep diaries, or objective measures, such as polysomnography (PSG) and actigraphy. Subjective sleep problems reported by adolescents and adults with ADHD include longer sleep onset latency, more night awakenings, higher daytime sleepiness and lower sleep efficiency (percentage of time spent asleep to time in bed) compared to adolescents and adults without ADHD [5–9]. Yet studies employing objective assessments often do not show as many sleep disturbances compared to subjective measures. Meta-analyses have reported no differences between adults with and without ADHD in sleep behaviours when measured by polysomnography and only identified a later sleep onset latency and lower sleep efficiency when measured by actigraphy [5, 6]. A meta-analysis reported no differences between adolescents with and without ADHD in sleep behaviours; however, only a few studies objectively measured sleep difficulties [9].

The different methods used to investigate ADHD and sleep problems each have unique limitations. Subjective reports may inflate sleep difficulties experienced. While PSG is the gold standard for measuring sleep [10], it is expensive and time-consuming. PSG also lacks ecological validity since it is typically recorded in a laboratory setting, and only one or two nights are measured. Although actigraphy overcomes some of the limitations of PSG, with the recent explosion of consumer wearable devices, there is increasing interest among researchers in the possible utility of consumer devices as acceptable alternatives to actigraphy in the measurement of sleep [11].

Wearable devices overcome the limitations of the traditional objective measures. Compared to PSG, wearable devices are more affordable, easier to set up, and can be worn at home, an ecologically valid environment. Compared to actigraphy, wearable devices often have wireless capability allowing them to be used for extended periods and often contain other sensors, such as heart rate. In small validation studies in generally healthy adults (no sleep disorder, mental disorder, or somatic illness), several consumer-grade devices have been shown to perform as well or even better than research-grade actigraphy devices [11].

Discrepancies between subjective and objective reports may also relate to subjective reports capturing problematic nights, which may not be captured in a single night’s sleep or by averaging objective measurements over several nights (such as average sleep duration, quality, and efficiency) [12]. While the mean differences in sleep that are typically reported for people with and without ADHD are relevant, sleep variability is perhaps of particular interest, given the behaviour variability that is common in ADHD [13, 14].

Previous research using actigraphy and sleep diaries (average of 14 days) has explored night-to-night variability in adolescents with and without ADHD (ages 12–14 years) [15]. For actigraphy, adolescents with ADHD were found to have greater variability for time in bed, sleep onset (time fell asleep) and offset (time woke up), and wake after sleep onset over two weeks than adolescents without ADHD. For sleep diary data, adolescents with ADHD had greater variability in sleep onset, sleep offset, sleep duration (total time spent asleep), sleep onset latency, sleep quality, and night wakings than adolescents without ADHD. By using wearable devices, we can collect nightly sleep data over longer periods of time for each participant and explore whether the variability of sleep features differs between adolescents (16 + years) and adults with and without ADHD.

Alongside sleep difficulties, up to 80% of individuals with ADHD also present with at least one additional co-occurring serious mental health problem, including anxiety and depression, in their lifetime [16–19]. Previous research using a Fitbit wearable device in adults with major depressive disorder found that when depressive symptoms worsened, sleep behaviours also changed (including lower sleep efficiency and more variable sleep onset, offset and duration) [20, 21]. Co-occurring anxiety and depression symptoms may exacerbate sleep difficulties in individuals with ADHD. For example, depressive symptoms may be associated with lower sleep stability and poorer sleep quality in individuals with ADHD.

Data collection using remote measurement technology (RMT), incorporating active and passive monitoring, may overcome many limitations of conventional research whilst adding minimal burden to the individual [22]. Active monitoring involves the participant completing tasks using a smartphone app (such as completing clinical questionnaires or speech tasks) or using a PC/laptop (cognitive tasks). Passive monitoring consists of ongoing data collection without active input from the participant; data can be collected on a wide range of variables, including a wearable device that can measure sleep, physical activity and heart rate. RMT now enables the monitoring of both sleep and the symptoms of psychiatric conditions for longer periods of time [20]. For example, data collection can involve participants wearing a wearable device to collect daily measures of sleep alongside answering multiple measures of clinical symptom questions (ADHD symptoms, depression and anxiety) at selected intervals. Wearable devices can also be worn for longer periods of time than typical objective measures, such as PSG and actigraphy.

We have developed a new RMT system, the ADHD Remote Technology (ART) system, for adolescents and adults (aged 16+) with ADHD. The ART system was used to monitor adolescents and adults with and without ADHD over a 10-week remote period that incorporated passive and active monitoring [23, 24]. Here, we focus on sleep features derived from a wearable device (Fitbit Charge 3) and clinical symptom questionnaires from the smartphone Active App. Our study addresses three research questions and hypotheses:

1. Are there differences in mean levels of sleep features (sleep duration, sleep onset and offset, and sleep efficiency) between individuals with and without ADHD over 10 weeks? We offer no directional hypothesis, as previous research using objective measures has produced contradictory findings.
2. Are there differences in the variability of sleep features (SD of sleep duration, SD of sleep onset and offset, and SD of sleep efficiency) between individuals with and without ADHD over 10 weeks? We hypothesise that individuals with ADHD will have more variability in sleep features compared to individuals without ADHD.
3. Across a 10-week remote monitoring period, are changes in clinical symptoms (anxiety and depressive symptoms) associated with changes in sleep features (sleep duration, sleep onset and offset, and sleep efficiency) measured over the previous two weeks? We hypothesise that higher anxiety and depressive symptoms will be associated with more sleep disturbances in both individuals with and without ADHD.

## Methods

### Participants

Between August and November 2020, we recruited 20 individuals with ADHD and 20 individuals without ADHD (a comparison group) aged 16–39. Participants were recruited from previous studies (where they had indicated that they were willing to be contacted regarding future research studies), via the Attention Deficit Disorder Information and Support Service (ADDISS), social media, King’s Volunteer email circular and the ‘Call for Participants’ website. Exclusion criteria were: (1) having psychosis, major depression, mania, drug dependency or a major neurological disorder (self-reported during screening call with the researcher); (2) any other major medical condition which might impact upon the individual’s ability to participate in normal daily activity; (3) pregnancy and (4) IQ of < 70. Participants in the ADHD group additionally met diagnostic criteria for ADHD using the Diagnostic Interview for ADHD in adults (DIVA), and participants in the comparison group could not meet diagnostic criteria for ADHD based on the self-report on Barkley Adult ADHD Rating scale on current symptoms (BAARS-IV) and Barkley ADHD functional impairment questionnaire.

### Ethical considerations

The study was approved by the North East – Tyne and Wear South Research Ethics Committee (REC reference: 20/NE/0034). Informed consent was obtained from participants before the assessments started. To maintain participant anonymity, their data are pseudonymised. Participant names were replaced with a code, and data collected from the apps, wearable devices and interviews are only associated with this code and stored separately from any personally identifiable information. Participants were compensated £30 after completion of the baseline sessions, £20 after the first remote active monitoring follow-up (week 5) and a further £50 at the study endpoint (week 10).

### Procedure

ART-pilot is an observational, non-randomised, non-interventional study using commercially available wearable technology and smartphone sensors. Participation does not change participants’ usual care or treatments.

Participants attended two virtual baseline sessions with a research worker using Microsoft Teams. The first virtual baseline session with the participants with ADHD included the administration of the following assessments: (1) the Diagnostic Interview for ADHD (DIVA) in adults [25] to confirm ADHD diagnosis, (2) vocabulary and digit span subscales from the Wechsler Abbreviated Scale of Intelligence (WASI-II) and Wechsler Adult Intelligence Scale (WAIS-IV), respectively, and (3) web-based REDCap baseline questionnaires. The second session was administered once participants had received their wearable device and smartphone by post, approximately a week after the first session. The second session included (1) administration of two cognitive tasks (Combined cued continuous performance test (CPT-OX) and Go/NoGo (GNG) task, and the Fast task) [24] and (2) a training session on the use of the wearable device, mobile sensors and a smartphone Active App. The participant also received a leaflet summarising key information (Participant Technology User Guide) and researcher contact details for future reference. Each session lasted for approximately 1.5 h. Participants in the comparison group were assessed similarly, except that instead of the full ADHD diagnostic interview, they completed the ADHD symptom and impairment questionnaire [26, 27].

Passive monitoring, using the smartphone and wearable device, started from the second baseline session and was ongoing for 10 weeks for each participant. Passive monitoring means that the data are collected automatically using the devices without any active input from the participant. Participants wore the wearable device for 10 weeks and had to carry the smartphone with them throughout this period.

Active monitoring took place on three occasions (weeks 2, 6, and 10), when the participants completed clinical symptom questionnaires on the smartphone Active App. Each participant was engaged in the study for 10 weeks.

### Sleep features

Passive data (data that do not require any conscious engagement from the participant) were collected continuously using a wearable device. Participants wore a Fitbit Charge 3 continuously for 10 weeks, tracking sleep using accelerometer sensors. We used Fitbit-derived sleep features previously described in an RMT study of patients with major depressive disorder [20, 21, 28]. Specifically, we chose four sleep features to reflect a participant’s sleep architecture and quality (Table 1). For each day, we focused on the longest continuous period of sleep, excluding shorter periods of sleep, such as diurnal naps.

**Table 1:** Fitbit-derived sleep features

	Sleep feature	Definition
Sleep architecture	Sleep duration	Daily total time spent asleep
	Sleep onset time	Time fell asleep (first recorded ‘non-awake’ sleep stage)
	Sleep offset time	Time woke up (last recorded ‘non-awake’ sleep stage)
Sleep quality	Sleep efficiency	Percentage of sleep duration to time in bed

### Measures

#### Baseline measures

##### Diagnostic interview for ADHD in adults [25]

Trained researchers conducted the Diagnostic Interview for ADHD in Adults (DIVA) to assess DSM-V criteria for adult ADHD symptoms and impairment. Each item is scored affirmatively if the behavioural symptom was present ‘often’ within the past 6 months.

##### Verbal IQ [29]

The vocabulary subtest of the Wechsler Abbreviated Scale of Intelligence (WASI-II) [29] was administered to all participants to estimate verbal IQ.

##### About you

The questionnaire includes items on demographics, medication history, service use and experience with technology.

##### Barkley Adult ADHD Rating scale on current symptoms (BAARS-IV) and Barkley ADHD functional impairment scale (BFIS) [27]

The BAARS-IV is an empirically developed self-report measure based on DSM diagnostic criteria for assessing current adult ADHD symptoms [26, 27]. The scale includes the 18 diagnostic ADHD symptoms (9 items in each domain of inattention and hyperactivity/impulsivity), with a reported alpha of 0.92 [26, 27]. The responses for each item are scored on a four-point rating scale (0 = ‘never or rarely’, 1 = ‘sometimes’, 2 = ‘often’ and 3 = ‘very often’). The 18 items in the scale are arranged so that symptoms associated with inattention are the odd-numbered items and the hyperactive-impulsive symptoms are even-numbered. Inattention symptoms and hyperactive-impulsive symptoms are scored separately. For each symptom, all items that scored 2 (‘often’) or 3 (‘very often’) are counted. For the present study, consistent with DSM-V criteria, a symptom count of five or more for inattention or hyperactivity-impulsivity was considered to meet DSM-V threshold for ADHD.

The BFIS is a 10-item scale used to assess the levels of functional impairments commonly associated with ADHD symptoms in five areas of everyday life: family/relationship; work/education; social interaction; leisure activities; and management of daily responsibilities. The BFIS has a reported alpha of 0.92 [30]. The responses for each item are scored on a four-point rating scale (0 = ‘never or rarely’, 1 = ‘sometimes’, 2 = ‘often’ and 3 = ‘very often’). To calculate the BFIS self-report total score, similar to the BAARS-IV, all items that scored 2 (‘often’) or 3 (‘very often’) are counted. For ART, the wording has been changed from “during the past 6 months” to “during the past 2 weeks”, as this allows the BAARS-IV and BFIS to be suitable active monitoring measures.

#### Active monitoring data

##### Generalized anxiety disorder-7 (GAD-7) [31]

The GAD-7 measures anxiety symptoms in the past two weeks and consists of 7 items scored from 0 (‘not at all’) to 3 (‘nearly every day’). The total score of all items can be used to evaluate the anxiety symptom severity of the participant. Scores of 5, 10, and 15 represent cut-points for mild, moderate and severe anxiety, respectively [31].

##### Patient health questionnaire depression scale (PHQ-8) [32]

The PHQ-8 measures depression symptoms in the past two weeks. The PHQ-8 consists of 8 items, scored on a range of 0 (‘not at all’) to 3 (‘nearly every day’). The total score of all items can be used to evaluate the depressive symptom severity of the participant. A total score of 0 to 4 represents no significant depressive symptoms. A total score of 5 to 9 represents mild depressive symptoms; 10 to 14, moderate; 15 to 19, moderately severe; and 20 to 24, severe. A PHQ-8 score of ≥ 10 is the recommended cut-off point for “clinically significant” depressive symptoms [32].

### Statistical analyses

Independent t-tests were run to examine group differences in age and WASI-II vocabulary subscale, and the chi-square test to test for a group difference in gender.

The statistical analyses were completed in four parts. First, we used linear mixed models to estimate mean differences in sleep features between participants with and without ADHD (research question 1). Each sleep feature was tested in a separate model where the outcome was the respective sleep feature. Each model included a binary group indicator (1 = with ADHD; 0 = without ADHD) and a participant-level random intercept to account for clustering of repeated assessments from the same participant over the 10-week period:

$$\:sleep\_featur{e}_{ij}={{\upbeta\:}}_{0}+{{\upbeta\:}}_{1}ADH{D}_{ij}+{u}_{0j}+{e}_{ij}$$

$$\:{u}_{0j}\sim\:N\left(0,{{\upsigma\:}}_{u0}^{2}\right)$$

$$\:{e}_{ij}\sim\:N\left(0,{{\upsigma\:}}_{e}^{2}\right)$$

Second, to explore variability in each sleep feature (research question 2), we calculated the standard deviation of each feature for each participant over a 10-week remote monitoring period. We used F-tests to compare the differences in variability between the two groups.

Third, we used linear mixed models to test associations of each sleep feature with anxiety and depression (research question 3). We extracted sleep information in the two weeks prior to clinical symptom questionnaires completed at weeks 2, 6, and 10. We restricted this set of analyses to participants with at least five days of sleep information per fortnight, following past evidence examining the reliability of daily diary and actigraphy data [33, 34]. Each sleep feature was tested in a separate model. Each model included an interaction term between the respective clinical symptom (anxiety symptoms and depressive symptoms) and group, a participant-level random intercept:

$$\:sleep\_featur{e}_{ij}={\beta\:}_{0}+{{\upbeta\:}}_{1}ADHD+{{\upbeta\:}}_{2}symptom{s}_{ij}$$

$$\:+{\:\beta\:}_{3}\left(symptoms\times\:\text{A}DHD\right)$$

$$\:+\:{u}_{0j}+{e}_{ij}\:$$

$$\:\:{u}_{0j}\sim\:N\left(0,{\sigma\:}_{u0}^{2}\right)$$

$$\:{e}_{ij}\sim\:N\left(0,{\sigma\:}_{e}^{2}\right)$$

Likelihood ratio tests were used to quantify whether including the interaction term (clinical symptom x ADHD group) results in a statistically significant improvement in model fit compared to the model without the interaction term. The threshold for statistical significance was *p* < 0.05. To estimate within- individual associations, all clinical symptoms were person-mean centred [35, 36] by subtracting from each measurement the participant’s mean value over the 10 weeks. Average marginal effects (AMEs) are used to report the within-person effect of clinical symptoms on sleep using the marginal effects package for R [37]. A marginal effect is the partial derivative of the regression equation with respect to each variable in the model for each unit in the data. The AME is the mean of these partial derivatives over the sample. In our case, AMEs represent the effect of a one-unit within-person difference in the clinical symptom (anxiety symptoms and depressive symptoms) on the respective sleep feature. The within-person association refer to differences over time from the individual’s mean symptom level (i.e., a one-unit change).

Data points that had four hours or more for time in bed were included. All sleep features, except sleep efficiency, were converted from time (hh: mm) to numeric variables for analysis. Histograms were computed to visualise normal distribution; most sleep variables represented a normal distribution and so were not transformed. Outliers were identified above the upper limit [38]; however, these did not affect the results and were therefore left in due to exploration of variability. *P*-values were corrected by using the Benjamini-Hochberg method [39] for multiple comparisons, and the significance level of the corrected *P* value was set to 0.05. Analyses were completed using R [40] and the lme4 [41] package.

## Results

The analytical sample comprised 40 participants (20 with ADHD and 20 without). For research question 3, we excluded one participant with ADHD due to missing information (< 5 nights of sleep information per fortnight or missing clinical symptom questionnaire data from two time points).

Table 2 presents the characteristics of the analytical sample at baseline. Participants with and without ADHD did not differ on gender, age, or verbal IQ, and both groups provided a similar number of nights of sleep data (median (IQR) for participants with ADHD = 62 (25); for participants without ADHD = 68 (10); t_(38)_= -1.9, *p* = 0.62). From 40 participants, there were 2,428 nights of sleep data.

**Table 2:** Demographics by group, with tests for differences between participants with and without ADHD

	Participants with ADHD (*n* = 20)	Participants without ADHD (*n* = 20)	*P*-value for group differences*
Gender, female %	75	75	1.00
Age, mean (SD)	27.49 (6.04)	27.79 (6.17)	0.88
WASI-II vocabulary subscale, mean (SD)	57.85 (7.53)	56.80 (8.35)	0.68
Number of nights of sleep data, median (IQR)	62 (25)	68 (10)	0.62

*Assessed using chi-square test (gender) and an independent sample t-test (age,* WASI-II*,* number of nights of sleep data)*

SD = standard deviation, IQR = interquartile range

### Are there differences in the mean level of sleep features between participants with and without ADHD over 10 weeks?

No significant differences emerged between the participants with and without ADHD on sleep features (Table 3).

**Table 3:** Sleep features in participants with and without ADHD

	Participants with ADHD (*n* = 20) mean (SD)	Participants without ADHD (*n* = 20) mean (SD)	Between group comparisons		
			t	*p*	Critical B-H value^*r*^
**Sleep architecture**					
Sleep duration (hh: mm)	07:20 (01:33)	07:37 (01:10)	-1.251	0.219	0.050
Sleep onset (hh: mm)	00:25 (02:01)	00:23 (01:43)	0.422	0.676	0.069
Sleep offset (hh: mm)	08:20 (01:52)	08:34 (01:37)	− 0.125	0.901	0.088
**Sleep quality**					
Sleep efficiency (%)	93.67 (4.23)	93.60 (3.67)	0.001	0.999	0.100

SD = standard deviation, ^r^Critical Benjamini-Hochberg value

*p* < 0.05

### Are there differences in the variability of sleep features between participants with and without ADHD?

Statistically significant group differences emerged for each measure of the variability of sleep features (Table 4). Participants with ADHD were more variable than participants without ADHD on sleep duration (SD of participants with ADHD: 1 h 33 min; SD of participants without ADHD: 1 h 10 min), sleep onset (SD of participants with ADHD: 2 h 2 min; SD of participants without ADHD: 1 h 43 min), sleep offset (SD of participants with ADHD: 1 h 50 min; SD of participants without ADHD: 1 h 37 min), and sleep efficiency.

**Table 4:** Variability of sleep features for participants with and without ADHD

	Participants with ADHD	Participants without ADHD	Between-group comparisons		
Sleep features			F statistic	*p*	Critical B-H value^*r*^
SD of sleep duration	1.55	1.16	0.56	< 0.001	0.006
SD of sleep onset	2.03	1.71	0.70	< 0.001	0.013
SD of sleep offset	1.86	1.62	0.76	< 0.001	0.019
SD of sleep efficiency (%)	4.23	3.67	0.75	< 0.001	0.025

SD = standard deviation, ^r^Critical Benjamini-Hochberg value

*p* < 0.05

### Across a 10-week remote monitoring period, are changes in clinical symptoms (anxiety and depressive symptoms) associated with changes in sleep features measured over the previous two weeks?

Table 5 presents within-individual associations of anxiety and depressive symptoms on each sleep feature. These estimates are based on pooled models combining participants with and without ADHD. We used likelihood-ratio tests to compare models with and without a group interaction term (e.g., ‘depressive symptoms’ × ‘ADHD group’). The within-individual association captures the effect of within-person symptom changes over time (i.e., deviation from the individual’s mean level). For all outcomes, we found no statistically significant interaction terms for clinical symptoms (depressive symptom or anxiety symptom) and all the sleep features. Supplementary Material 1 presents the within-individual association of anxiety and depression on each sleep feature, based on separate and pooled models combining participants with and without ADHD.

For sleep architecture, within-individual associations for anxiety symptoms and depressive symptoms were non-significant. For sleep quality, within-individual associations for anxiety symptoms and depressive symptoms were non-significant.

**Table 5:** Within-individual associations of co-occurring anxiety symptoms and depressive symptoms over the previous two weeks on sleep difficulties, pooled for participants with and without ADHD

	Anxiety symptoms			Depressive symptoms		
	AME [95% CI]	Likelihood-ratio test		AME [95% CI]	Likelihood-ratio test	
		*P*-value	Critical B-H value^*r*^		*P*-value	Critical B-H value^*r*^
**Sleep architecture**						
Sleep duration	0.01 [-0.03, 0.06]	0.093	0.031	0.01 [-0.04, 0.06]	0.806	0.081
Sleep onset	0.04 [-0.04, 0.11]	0.631	0.063	0.04 [-0.04, 0.12]	0.101	0.038
Sleep offset	0.05 [-0.04, 0.13]	0.917	0.094	0.04 [-0.05, 0.13]	0.168	0.044
**Sleep quality**						
Sleep efficiency (%)	− 0.09 [-0.21, 0.02]	0.390	0.056	− 0.00 [-0.13, 0.12]	0.774	0.075

* *p* < 0.05, ^r^Critical Benjamini-Hochberg value

CI = confidence interval; AME = average marginal effects; LR test = Likelihood-ratio test of the interaction term, comparing the model with main effects only to a model that additionally included the interaction term (clinical symptom × ADHD group)

All tested separately

## Discussion

In a 10-week remote monitoring study of sleep in individuals with and without ADHD using a wearable device, we found that it was sleep variability, rather than the amount or quality of sleep, that distinguished individuals with and without ADHD. Whereas participants with ADHD had a more variable sleep duration, sleep onset and offset, and sleep efficiency, their mean values on these measures were similar to individuals without ADHD. Inconsistency and high variability in behaviour, as well as cognitive performance, is a hallmark of ADHD [13, 14] and our findings suggest this characteristic extends also to sleep features.

### Main findings

Based on RMT collected continuously over a 10-week period, we observed no mean differences in sleep features between individuals with and without ADHD: mean sleep duration, mean sleep onset and offset, and mean sleep efficiency were comparable between the groups. These findings are consistent with previous studies that have used PSG to measure sleep in individuals with and without ADHD [5, 6]. However, our finding of no mean group differences in sleep efficiency is inconsistent with studies that have used actigraphy to measure sleep with individuals with and without ADHD [5, 6].

In contrast, compared to participants without ADHD, those with ADHD had more variable sleep duration, onset and offset, and efficiency. This suggests that the people with ADHD had less regular sleep habits and characteristics, and varied more – night to night across the 10-week remote monitoring period – in their sleep features. Participants without ADHD spent a more consistent amount of time spent asleep, and had a more consistent sleep onset and offset, and percentage of time spent asleep while in bed each night. These findings support a previous study that found adolescents with ADHD had more night-to-night variability for sleep onset and offset (measured through actigraphy and sleep diaries) than adolescents without ADHD [15]. Variability in sleep features may link to the core symptoms of ADHD. Symptoms of ADHD, such as difficulties with attention, restlessness in the evening and poor organisation [42], may result in more variable sleep behaviours in individuals with ADHD compared to those without ADHD. Inconsistency and high variability in behaviours have also been found in other aspects of ADHD, such as cognitive performance [13, 14].

This variability in sleep features could potentially explain the discrepancy found between subjective reports and objective measures of sleep with adolescents and adults with ADHD [5, 6, 9]. Subjective reports may indicate more sleep difficulties as they capture those problematic nights where the individual may have a later sleep onset or a shorter sleep duration. Objective measures may indicate fewer sleep difficulties when the mean values are used to describe the sleep behaviour, potentially hiding the problematic nights that are reported by individuals with ADHD.

We found no evidence of a relationship between clinical symptoms of anxiety and depression, measured over the previous two weeks, with sleep (sleep architecture and sleep quality) in either individuals with or without ADHD. Including ‘group’ as an interaction term in the model did not improve the fit, suggesting that group affiliation (with ADHD vs. without ADHD) did not modify the association between the co-occurring clinical symptoms of anxiety and depression with the sleep features. A previous RMT study that also used RADAR-base and Fitbit wearable devices over two years found that when depressive symptoms worsened in adults with major depressive disorder, sleep behaviours also worsened [20]. It is possible that our shorter monitoring period of 10 weeks, compared to a longer monitoring period such as two years in a previous study on depression [20], was insufficient to capture substantial changes in anxiety and depressive symptoms. This emphasises the need for longer-term monitoring studies.

### Limitations

Although appropriate for a pilot study, our sample size was small, and these findings should be replicated in larger samples. However, despite the modest number of participants, our repeated measures design allowed us to use all assessments collected over 10 weeks, providing a rich data on 2,428 nights of sleep. In our sample, we included both adolescents and adults (aged 16–39). Different social factors (e.g., work patterns, school schedule) could influence sleep patterns and behaviours. Importantly for our analyses, however, the groups were matched on age. Furthermore, by using linear mixed models in Research Questions 2 and 3 we were able to explore night-to-night variability (i.e., within-individual changes) for each participant. As we focused on sleep problems in a representative sample of people with ADHD, we did not screen the participants for specific sleep disorder diagnoses. Future larger studies could investigate the impact of specific co-occurring conditions. Another limitation is that the data were collected during the COVID-19 pandemic. Due to pandemic-related restrictions, sleep behaviours may have differed compared to pre-pandemic sleep behaviours [43]. However, data for both groups (with and without ADHD) were collected during the pandemic, and therefore, both groups would have been affected by the restrictions. Finally, although participants were instructed to wear the Fitbit Charge 3 continuously for 10 weeks (only removing it to charge and shower), there was some variability in adherence. However, participants with and without ADHD had a similar number of nights recorded for sleep behaviours.

### Future research

In this study, participants with ADHD were either medication naïve or not currently taking medication for their ADHD. A meta-analysis of nine studies found that stimulant use was associated with moderately reduced total sleep duration, delayed sleep onset, and slightly to moderately decreased sleep efficiency in children and adolescents with ADHD [44]. Therefore, it is unclear whether we can generalise our findings to individuals with ADHD who are currently taking ADHD medication; this should be explored further. We are currently conducting a large remote monitoring study on adults with ADHD – the ADHD Remote Technology study of cardiometabolic risk factors and medication adherence (ART-CARMA) [45]. ART-CARMA utilises the ART system and incorporates both active (questionnaires, cognitive tasks, speech samples) and passive monitoring (smartphones and a novel wearable device, the EmbracePlus). In ART-CARMA, we monitor adults with ADHD both before and after initiation of ADHD medication treatment, which allows us to examine the effects of medication on sleep in adults with ADHD.

## Conclusions

Individuals with ADHD had more variable sleep patterns compared to those without ADHD. Variable sleep behaviours in individuals with ADHD may link to the core symptoms of ADHD, such as difficulties with attention, restlessness in the evening, and poor organisation. Future research should monitor sleep over longer periods to capture variability in sleep behaviours. This study also supports the need for interventions that collect real-time real-world daily data to capture problematic nights and to identify potential modifiable behaviours (i.e., reasons for inconsistent bedtime) that could reduce variability in sleep in individuals with ADHD.

## Electronic supplementary material

Below is the link to the electronic supplementary material.

Supplementary Material 1

## Data Availability

The data supporting this article is not openly available but may be shared on request following a formal review process by the chief investigator and co-investigators.

## Abbreviations

ADHD: Attention deficit hyperactivity disorder
AME: Average marginal effect
ART-CARMA: ADHD Remote Technology study of cardiometabolic risk factors and medication adherence
ART: ADHD Remote Technology
BAARS-IV: Barkley adult ADHD rating scale
DIVA: Diagnostic interview for ADHD
GAD-7: Generalized anxiety disorder
PHQ-8: Patient health questionnaire
PSG: Polysomnography
RADAR: Remote Assessment of Disease and Relapses
RMT: Remote measurement technology
WAIS-IV: Wechsler adult intelligence scale
WASI-II: Wechsler abbreviated scale of intelligence
