# Supplementary material for: A 10-week remote monitoring study of sleep features and their variability in individuals with and without ADHD
Source: BMC Psychiatry. 2025 Mar 27;25:294. doi: 10.1186/s12888-025-06729-z (PMC11951673; doi:10.1186/s12888-025-06729-z)
Supplement: Supplementary file 1 — Supplementary Material 1 [file 12888_2025_6729_MOESM1_ESM.docx]

## Supplementary material

**Supplementary material 1.** Within-individual associations of anxiety and depressive symptoms over the previous two weeks on sleep difficulties.

Notes. The table present the average marginal effects (AMEs) for the ADHD and control groups separately. The AME represent the change in sleep feature associated with a one-unit increase in clinical symptom (anxiety or depressive symptoms). Estimates for participants with and without ADHD were derived from a single model using Group-Average Marginal Effects (G-AME) calculated using the marginal effects package for R (Arel-Bundock, 2023). Each model included the interaction of group (with ADHD vs. without ADHD) with the respective clinical symptom.

|  | **Anxiety symptoms** | | | | |
| --- | --- | --- | --- | --- | --- |
|  | **Participants**  **with ADHD** | **Participants**  **without ADHD** | **Pooled** | | |
|  | **AME**  **[95% CI]** | **AME**  **[95% CI]** | **AME**  **[95% CI]** | **Likelihood-ratio test** | |
|  |  |  |  | ***p-value*** | **Critical B-H value^r^** |
| **Sleep architecture** |  |  |  |  |  |
| Sleep duration | .05  [-.01, .11] | -.03  [-.09, .04] | .01  [-.03, .06] | .093 | .031 |
| Sleep onset | .02  [-.08, .12] | .06  [-.05, .17] | .04  [-.04, .11] | .631 | .063 |
| Sleep offset | .05  [-.06, .17] | .04  [-.08, .17] | .05  [-.04, .13] | .917 | .094 |
| **Sleep quality** |  |  |  |  |  |
| Sleep efficiency (%) | -.04  [-.20, .11] | -.14  [-.32, .03] | -.09  [-.21, .02] | .390 | .056 |
|  | **Depressive symptoms** | | | | |
| **Sleep architecture** |  |  |  |  |  |
| Sleep duration | .02  [-.03, .07] | .00  [-.08, .09] | .01  [-.04, .06] | .806 | .081 |
| Sleep onset | -.02  [-.10, .05] | .10  [-.03, .24] | .04  [-.04, .12] | .101 | .038 |
| Sleep offset | -.02  [-.11, .07] | .01  [-.05, .26] | .04  [-.05, .13] | .168 | .044 |
| **Sleep quality** |  |  |  |  |  |
| Sleep efficiency (%) | .02  [-.15, .11] | .01  [-.20, .23] | -.00  [-.13, .12] | .774 | .075 |

** p<0.05, **p<0.01, ^r^Critical Benjamini-Hochberg value*

*CI = confidence interval; AME = average marginal effects; LR test = Likelihood-ratio test of the interaction term, comparing the model with main effects only to a model that additionally included the interaction term (clinical symptom × ADHD group).*

*All tested separately*
